# Connections between competencies and abilities in female artistic gymnasts and handball players

**DOI:** 10.3389/fspor.2025.1681743

**Published:** 2025-11-14

**Authors:** Vivien Wehovszky, Csaba Nagykáldi, Zsuzsanna Kalmár, Csaba Ökrös

**Affiliations:** 1 School of Doctoral Studies, Hungarian University of Sports Science, Budapest, Hungary; 2 Department of Gymnastics, Rhythmic Gymnastics, Dance and Aerobics, Hungarian University of Sports Science, Budapest, Hungary; 3 Department of Sport Games, Hungarian University of Sports Science, Budapest, Hungary

**Keywords:** competence, physical abilities, female artistic gymnastics, handball players, athlete development

## Abstract

Competencies play a pivotal role in addressing challenges in various domains of life, including physical education and sports, where their complexity and diversity are particularly emphasized. The primary aim of this study is to explore the development of sport-specific competencies from the perspective of physical abilities. A secondary aim is to investigate potential differences in competency profiles of different sports disciplines. To this end, the following hypotheses were proposed: Fundamental physical abilities are hypothesized to serve as prerequisites for the development of competencies. It is expected that these physical abilities will exhibit strong correlations with competencies understood as acquired skills. It is further hypothesized that there are measurable differences in competencies understood as associated with physical abilities among athletes from different sports, specifically between female gymnasts and handball players in our sample. Physical abilities were assessed through self-perception using a Likert scale, not through objective performance tests. To test these hypotheses, questionnaire-based assessments were employed. Physical abilities were measured by using the Actual Fit Efficacy (AFE) test, which evaluates ten distinct physical attributes. Validity was demonstrated through a correlation of R = 0.78 with the physical factor of the Competitor Self-Assessment Method (CSAM) meeting standard validity criteria. Competencies were assessed by using the CSAM, which includes 12 Likert-type scales. A test-retest analysis revealed high correlation coefficients for all scales (*p* < 0.001), confirming reliability. Criterion validity was established through comparisons of performance under low- and high-achievement conditions, with significantly higher scores observed during high-performance scenarios (*p*-values ranging from <0.05 to <0.001).

**Results:**

Among the correlations between physical abilities and competencies, 22.5% were statistically significant (*p* < 0.05) for the artistic gymnasts, supporting the first hypothesis and underscoring the foundational role of physical abilities in the development of competencies. In contrast, only 7.5% reached significance for the handball players. For gymnasts, key competencies included *physical condition*, *work ability*, *activity*, *competition attitude*, *performance expectation*, and *satisfaction*. In contrast, handball players demonstrated stronger associations with *work ability* and *activity*. These findings suggest the need for targeted competency development strategies tailored to the specific demands of each sport to enhance athletic performance.

## 1 Introduction

### 1.1 Theoretical background and interpreting competencies

The concept of competence is widely used and applied in a variety of interpretive contexts (1, 2). As a result, its description and annotation are multifaceted. Competence is referred to as knowledge, expertise, or specialized knowledge—representing theoretical understanding on the one hand (3), and abilities, proficiencies, or skills on the other, relating to the execution of practical tasks (4). An individual possessing the required competencies is considered competent—qualified to carry out specific tasks—whereas someone with low or insufficient competencies is deemed unfit or unqualified to perform those tasks (2, 5). Competencies are complex characteristics or sets of attributes that integrate knowledge, attitudes, behavioral tendencies, and essential psychological and physical capabilities in order to enable an individual to perform a given activity effectively and successfully (1, 6). This complexity encompasses both personal factors (e.g., motivation, self-confidence) and contextual conditions that facilitate successful functioning (3).

### 1.2 Physical abilities

In this study, physical abilities are defined as foundational, trainable attributes that underpin sport performance—typically encompassing strength, speed, endurance, flexibility/mobility, and coordination/skill-related capacities. In the sport context, these abilities act as key physiological and neuromuscular resources that enable the execution of sport-specific tasks and regulate fatigue, efficiency, and injury risk (7, 8). These foundational abilities form the physiological basis upon which sport-specific competencies can be developed and expressed. In the present research, these abilities were assessed through athletes' self-reported ratings on a five-point Likert scale, reflecting their extensive training and competition experience (9).

### 1.3 Key domains of competence

Leadership and management have placed particular emphasis on the study of competencies. Early contributions from leadership psychology laid the foundation for understanding competencies through task-oriented and relationship-oriented models (10), followed by multidimensional leadership theories (11). Over time, the body of knowledge expanded to include competencies essential for organizational governance across sectors such as industry, commerce, finance, public administration, military leadership, and broader societal areas including education, politics, and sport pedagogy (2, 4).

The British Association of Sport and Exercise Sciences (BASES) developed a competence profile, conceptualizing competence as a foundational and theoretically coherent framework for research and application in the field (12). In summary, psychological traits and interpersonal relationships form the subjective, human side of competence, while the specific characteristics and tools of each profession represent the objective conditions for effective functioning (1).

Depending on the activity, various lists of competencies are proposed to support efficient and successful work performance (3). In public education, international assessments such as the PISA surveys are widely used to evaluate students’ levels of knowledge and their ability to apply it (13). These assessments are conducted annually and serve as indicators of educational quality. They evaluate subjects taught in schools, background knowledge, and their practical applications. At the core of applicable knowledge are competencies such as reading comprehension, mathematical reasoning, and scientific critical thinking. Results have shown that competency levels fluctuate, and in some areas (e.g., creative thinking), there are notable deficiencies. As a response, numerous competency development programs have been proposed to address these gaps. These initiatives emphasize the importance of applied skills over theoretical knowledge (14, 15).

Several studies in the field of sports and physical education have examined coaching styles, highlighting the pedagogical competencies of teachers and instructors that play a crucial role in both athlete preparation and performance outcomes (6, 16). Coaching and sports teaching activities have been characterized by levels of communication, democratic and autocratic tendencies, social support, interpersonal feedback, and care. These factors are closely linked to athletes’ developmental potential (17). Just as the teacher–student relationship is fundamental to effective physical education, the coach–athlete relationship is equally essential for fostering progress and achieving performance goals (18).

In the context of sports and physical education, motor and psychomotor competencies are of primary importance, forming the basis for motor learning, coordination, and the execution of movements (19, 20).

## 2 Materials and methods

### 2.1 Research aim and objectives

The aim of our research is to evaluate the correlations between 12 psychosocial competencies and 10 self-perceived physical abilities in artistic gymnastics and handball athletes, thereby enhancing the understanding of athletic effectiveness (6). Our primary objective is to assess the key competencies that play a critical role in sports, and to examine the fundamental physical abilities that support the development of these competencies. The evaluation of physical abilities is essential, as athletic competencies are built upon various physical and psychomotor skills, which significantly contribute to achieving optimal performance (21).

General objective:

1. To evaluate the correlations between 12 psychosocial competencies and 10 self-perceived physical abilities in artistic gymnastics and handball athletes.

Specific objectives

1. To compare the correlation patterns between artistic gymnastics and handball athletes.
2. To identify which psychosocial competencies show the strongest associations with specific physical abilities.
3. To examine whether sport-specific movement characteristics explain differences in correlation structures.

Several studies strongly emphasize the combined influence of physical and psychological factors on sports performance. Abbott and Collins (22) highlight the multidimensional factors that influence performance, while Vaeyens, Lenoir, Williams, and Philippaerts (23) suggest that both physical characteristics and cognitive competencies collectively determine athletic development. Research on sport-specific competency requirements (24) has demonstrated that, in most cases, differing sports exhibit distinct correlation patterns.

Rationale for the joint examination of competencies and physical abilities. It is crucial to investigate psychological competencies and physical abilities together because elite sport performance emerges from their continuous interaction rather than from isolated factors. Integrated models of athletic development consistently highlight that mental skills such as self-regulation, confidence, and attentional control amplify the effects of physical preparation, while high levels of strength, endurance, and coordination provide the foundation for applying those mental skills under pressure (22, 25). Examining these domains in combination allows us to identify how psychological competencies can either potentiate or limit the expression of physical capacities during competition, thereby offering a more holistic and ecologically valid understanding of athletic effectiveness (21, 23).

### 2.2 Hypothesis

We hypothesize that if data are available on both competencies and levels of specific physical abilities, the interrelatedness of these variables can be examined. We expect that these relationships will be characterized by high correlation coefficients, indicating the physical ability foundations of various competencies. Additionally, we expect that different competencies will exhibit distinct correlation patterns across various sports, enabling targeted sport-specific comparisons. Uncovering these relationships may contribute to the development of personalized training and development programs for athletes, considering both competency structures and physical ability characteristics.

**H1:** There is a significant positive correlation between athletes' competencies and the specified physical abilities.

**H2:** In case of the examined sports (artistic gymnastics and handball), the correlation patterns of competencies will show differences meaning that each sport emphasizes distinct types of competencies.

### 2.3 Research samples

The study investigates two female groups from distinctly different sports: members of the Hungarian national female's artistic gymnastics team (*N* = 14, average age: 19–20 years) and elite female handball players who are members of a handball academy (*N* = 20, aged 18–19 years). The groups are homogeneous in terms of gender and age. Their differing sport-specific characteristics make them highly suitable for comparative analysis.

The differences in movement, decision-making, and coordination demands of two sports serve as the foundation for differing competency–ability correlation patterns. For example, athletes in artistic gymnastics emphasize precision, concentration, and body awareness, whereas handball players have placed more emphasis on teamwork, rapid reaction, and decision-making competencies (24, 26).

The participants were female elite athletes recruited from the Hungarian National Artistic Gymnastics Team (*N* = 14) (mean age = 18.5 years) and from professional Handball Academies (*N* = 20) (mean age = 19.5 years). Data collection took place in the spring of 2024, and completing the questionnaires required an average of approximately 12 min. Before participation, all athletes received detailed instructions and clarifications regarding the concepts used in the questionnaire.

**Inclusion criteria:** at least five consecutive years of regular training in the respective sport, current active competitive status, and absence of any acute injury. **Exclusion criteria:** current musculoskeletal injury or any chronic illness that could affect performance. All participants engaged in a minimum of four training sessions per week, each lasting 2–4 h.

### 2.4 Ethical approval

The research was reviewed and approved by the Ethics Committee of the Hungarian University of Sports Science on March 20. 2024. Approval ID: TE-KEB/No01/2024.

### 2.5 Applied methods

As a first step, we define ten key differences between artistic gymnastics and handball. Based on these distinctions, we expect to observe corresponding differences in their correlation patterns.

First, we systematically describe ten fundamental sport-specific characteristics—such as type of movement chain (closed vs. open skill), the presence or absence of an opponent, and the nature of performance evaluation—that clearly distinguish artistic gymnastics from handball. These predefined characteristics provide the theoretical rationale for our hypotheses. Because the two sports demand different motor, cognitive, and organizational skills, we predict that the patterns of correlation between psychosocial competencies and self-perceived physical abilities will also differ, reflecting the sport-specific requirements (Table 1).

**Table 1:** Distinct characteristics of artistic gymnastics and handball.

Athlete competencies	Characteristics	Artistic gymnastics	Handball
1.	Subject and object of execution^a^	Subject and object are same (immanent sport)	Subject (player) and object (the ball) are separated
2.	Type of movement chain^b^	Closed-chain movement skills	Open-chain movement skills
3.	Movement program	Prescribed choreographed	Free, variable movement program (situation –dependent)
4.	Coordination	Coordination of whole body and body parts	Situation-dependent, individual and team coordination,
5.	Nature of performance	Individual, partially choreographed	Teamwork, individual execution
6.	Work organisation	Stable, precise, individual	Team play, situation-dependent, individual solutions
7.	Performance dynamics	Sustained, individual, peak performance	Changing situations, quick decisions, tactical decision-making
8.	Mental processes	Imagination, internal visualisation	Situation recognition and creation, decision-making
9.	Role of the opponent	No present opponent	Opponents obstruct actions
10.	Performance evaluation	Judging scores	Based on goals and referee decisions

a *Subject and Object of Execution—*The performer (subject) and the implement or environment directly involved in the movement (object). In immanent sports the subject and object coincide (e.g., the gymnast's own body); in mediated sports the subject manipulates an external object, such as a ball (27).

b **Closed-skill** sports: the environment is stable and predictable, movements are pre-planned and repetitive, and athletes perform at their own pace. Emphasis is on technical precision and consistency. Examples include artistic gymnastics, figure skating, weightlifting, and shooting. **Open-skill** sports: the environment is dynamic and unpredictable, movements are situation-dependent, and athletes must constantly adapt to opponents and changing play conditions. Success depends on quick decision-making, rapid reactions, and tactical flexibility. Examples include handball, soccer, basketball, and fencing (27).

The **Competitor Self-Assessment Method (CSAM**) questionnaire—previously developed and validated—is applied in this study. It includes 5-point Likert scale items evaluating 12 different competencies: (1) Physical condition, (2) work ability, (3) activity, (4) mood, (5) concentration, (6) movement regulation, (7) technical level, (8) training motivation, (9) self-confidence, (10) competition attitude, (11) performance expectation, and (12) satisfaction (28). To ensure a clear understanding of the competencies, the relevant concepts were defined and provided to the athletes for review prior to completing the test. The test has been found to be reliable based on item analysis and valid based on criterion-related validity (28). It has previously been used to assess gymnasts' self-perceived competencies (29).

To assess physical abilities, the **Actual Fit Efficacy** test **(AFE)** is used, in which athletes provide self-reported ratings of their own abilities, which employs 5-point Likert scale ratings to evaluate subcomponents of physical performance. Physical abilities, defined here as athletes' self-perceived physical capacities, were assessed through self-reported ratings on a 5-point Likert scale rather than through objective performance tests. Given that the participants were experienced athletes with extensive training and competition backgrounds, it is assumed that they can consciously and accurately evaluate their physical capacities based on their substantial practice and competition experience. The test includes the following items: (1) physical (health-related) well-being, (2) single-effort capability, (3) movement speed, (4) endurance in work, (5) explosive power, (6) joint flexibility, (7) sustained effort capacity, (8) dexterity, (9) muscle relaxation, and (10) sustained speed. The test's reliability is supported by a Cronbach's alpha of 0.79 and a Spearman-Brown coefficient of 0.71. Validity has been demonstrated through parallel test correlations (R = 0.67–0.88) across six handball teams (9).

### 2.6 Statistics

In total, 12 × 10 = 120 correlations were computed between 12 psychosocial competencies and 10 self-perceived physical abilities in each sport. Pearson coefficients (two-tailed) were used. Given the exploratory, pilot design and the small sample sizes (gymnastics *N* = 14; handball *N* = 20), no correction for multiple comparisons, was applied to avoid excessive Type II errors. Therefore, the reported associations should be interpreted as preliminary and hypothesis-generating, serving as a starting point for future research with larger samples.

## 3 Results

To evaluate the data, we chose to analyse the correlation coefficients which allows us to map the relationships’ structure between competencies and physical abilities. The table represents the correlations between 12 competencies and 10 physical sub-abilities in both sport-specific groups (artistic gymnastics and handball), resulting in a total of 120 correlation links for each group.

This structure enables the correlation database to yield both quantitative and qualitative insights into the patterns of relationships between competencies and physical abilities.

In the case of artistic gymnastics group, out of all 120 correlations, 27 of 120 correlations (22.5%) showed statistically significant relationships (*p* < 0.05), including 4 correlations that had *p*-values between 0.05; 0.01; 0.001, which may indicate potential trends but do not meet conventional significance criteria. Together, these account for 33.3% of the total correlation field. For the handball group, a similar ratio was observed; 9 of 120 correlations (7.5%) showed statistically significant relationships (*p* < 0.05).

Because of the small sample sizes and the exploratory design, these *p*-values and correlation coefficients should be interpreted cautiously. 95% confidence intervals were not calculated, but are expected to be wide, reflecting substantial statistical uncertainty. The results are therefore descriptive and hypothesis-generating, providing signals for future sport-specific comparisons.

These proportions show that physical abilities are significantly intertwined with competencies. Competencies are not only “built” on physical abilities, but as previously mentioned, are also the result of a long learning and practice process and therefore have cognitive components. However, the aim of this study is to demonstrate how physical abilities are connected to specific competencies, since sporting activity is fundamentally a physical activity.

Based on the frequency of correlations per competency, four zones were defined: high frequency (5–7 cases per competency), medium frequency (2–4 cases), low frequency (1–2 cases), and zero frequency. This classification allows for an evaluation of not just the existence of specific correlations, but also of their relative frequency at the level of each competency.

First, we contrast the competencies with high frequency (5–7 significant correlations) between the artistic gymnastics and handball sports. Based on Table 2, a surprising result is that there are no common high-frequency correlations between abilities and competencies in the two sports under study. This finding can lead to qualitative conclusions. It suggests that although both sports are fundamentally based on physical activity, there are significant differences in the way competency systems function and in the physical foundations of these competencies.

**Table 2:** Correlation patterns between competencies and abilities—in the case of the Female's gymnastics and Female's handball teams.

Results: Competencies	Gymnastics national team (*N* = 14)	Handball youth team (*N* = 20)
Physical condition	**Physical well-being (*r* = 0.723): *p* < 0.01; CI: 0.31–0.91 **Single-effort capability (*r* = 0.674): *p* < 0.01; CI: 0.22–0.89 *Endurance work (*r* = 0.620): *p* < 0.02; CI: 0.13–0.87 *Explosive power (*r* = 0.63): *p* < 0.02; CI: 0.15–0.87 **Dexterity (*r* = 0.697): *p* < 0.01; CI: 0.26–0.90 ***Sustained speed (*r* = 0.779): *p* < 0.001; CI: 0.42–0.93	–
Work Ability	*Physical well-being (*r* = 0.639): *p* < 0.02; CI: 0.16–0.88 **Endurance work (*r* = 0.764): *p* < 0.01; CI: 0.36–0.39 *Explosive power (*r* = 0.545): *p* < 0.05; CI: 0.01–0.83 **Sustained effort capacity (*r* = 0.687): *p* < 0.01; CI: 0.24–0.90 **Dexterity (*r* = 0.785): *p* < 0.01; CI:0.40–0.94	*Movement speed (*r* = 0.452): *p* < 0.05; CI: −0.01–0.74 ***Joint flexibility (*r* = 0.792): *p* < 0.001; CI: 0.52–0.92 **Muscle relaxation (*r* = 0.637): *p* < 0.01; CI: 0.23–0.85
Activity	**Physical well-being (*r* = 0.706): *p* < 0.01; CI: 0.26–0.90 *Single-effort capability (*r* = 0.561): *p* < 0.05; CI: 0.01–0.83 *Sustained effort capacity (*r* = 0.624): *p* < 0.05; CI: 0.13–0.87 **Dexterity (*r* = 0.735): *p* < 0.01; CI: 0.31–0.91 **Sustained speed (*r* = 0.673): *p* < 0.01; CI: 0.220–0.89	*Endurance work (*r* = 0.481): *p* < 0.05; CI: 0.01–0.76 **Joint flexibility (*r* = 0.596): *p* < 0.01; CI: 0.17–0.83 **Muscle relaxation (*r* = 0.639): *p* < 0.01; CI: 0.23–0.86
Mood	–	–
Concentration	–	–
Movement Regulation	–	–
Technical Level	*Endurance in work (*r* = 0.604) *p* < 0.05; CI: 0.10–0.87	*Muscle relaxation (*r* = 0.476): *p* < 0.05; CI: 0.03–0.75
Training Motivation	**Sustained speed (*r* = 0.692) *p* < 0.01; CI: 0.22–0.89	*Endurance work (*r* = 0.51): *p* < 0.05; CI: 0.01–0.75
Self-confidence	–	–
Competition Attitude	**Single-effort capability (*r* = 0.674): *p* < 0.01; CI: 0.22–0.89 *Movement speed (*r* = 0.638) *p* < 0.02; CI: 0.16–0.88	–
Performance Expectation	*Physical well-being (*r* = 0.589) *p* < 0.05; CI: 0.06–0.86 *Muscle relaxation (*r* = 0.553) *p* < 0.05; CI: 0.84–0.04	*Sustain effort capacity (*r* = 0.468): *p* < 0.05; CI: 0.01–0.75
Satisfaction	**Physical well-being (*r* = 0.66) *p* < 0.01; CI: 0.21–0.88 **Single-effort capability (*r* = 0.773) *p* < 0.01; CI: 0.39–0.93 *Explosive power (*r* = 0.559) *p* < 0.05; CI: 0.01–0.84 *Joint flexibility (*r* = −0.566) *p* < 0.05; CI: −0.84–−0.02 *Sustained speed (*r* = 0.545) *p* < 0.05; CI: 0.00–0.83	–

Physical (health-related) well-being: refers to the general state of physical health and fitness that supports optimal performance and recovery. Single-effort capability: describes the ability to produce maximal force or performance in a single attempt (e.g., jump, throw). Movement speed: the ability to perform movements quickly in response to stimuli or during specific motor tasks. Endurance in work: represents the capacity to maintain performance during prolonged physical activity. Explosive power: combines strength and speed to generate force rapidly, essential for jumps, sprints, or throws. Joint flexibility: the range of motion available in a joint, enabling efficient and injury-free movement. Sustained effort capability: the ability to perform submaximal effort repeatedly over time without excessive fatigue. Dexterity: the skillful and precise coordination of movements, especially those requiring fine motor control. Muscle relaxation: the ability to consciously release tension and alternate between contraction and relaxation efficiently. Sustained speed: the ability to maintain a high movement velocity over an extended period.

r = Pearson correlation coefficient. CI = 95% confidence interval. Significance levels: *p* < 0.05: significant; *p* < 0.01: highly significant; *p* < 0.001: extremely significant.

* Significant.

** Highly significant.

*** Extremely significant.

According to Table 2, the artistic gymnastics group is primarily characterized by the competencies of physical condition, work ability, activity, competitive attitude, performance expectations, and satisfaction, along with their related abilities. These factors are associated with individual physical exertion and endurance-type components. The results reflect the nature of gymnastics, where athletes require constant self-monitoring, precise technical execution, and independent preparation. Furthermore, the emphasis on satisfaction and performance expectations supports the widely accepted notion that gymnastics, including artistic gymnastics, belongs to the category of sports characterized by strong internal motivation and self-reflection (30).

In contrast, the handball group's competency profile is marked by work ability and activity. This result indicates that, for handball players, these two factors are closely linked to physical abilities and play a fundamental role in determining performance during matches.

The differences between the two sports discussed earlier reinforce the findings of other research that different sports require distinct competencies and ability profiles from athletes (22, 31). Accordingly, this study provides conclusions that are not only relevant to general theoretical models but also applicable to specific sports practices.

Based on the research results, it can be stated that a single physical ability may be linked to multiple competencies. Therefore, rather than one-to-one correspondence, these abilities serve as multidirectional background functions. This complex interrelationship faithfully reflects how physical attributes play a multifaceted role within competency systems and how this varies by sport. According to the results, in the case of artistic gymnasts, the most frequently associated competencies are single effort capability, sustained speed and physical well-being (4–5 occurrences), followed by endurance in work, explosive power and dexterity (3). These characteristics clearly reflect the nature of gymnastics as a sport that requires technical precision, control, and the execution of high-intensity elements and jumps during long training sessions. Sustained effort capability shows only two instances of high correlation, while movement speed, muscle relaxation and join flexibility appear in only one case. This suggests that in artistic gymnastics, endurance, precise muscle control, and optimal movement coordination are more important than excessive joint flexibility, which can compromise a gymnast's ability to maintain body control during aerial movements.

In contrast, the correlation structure among handball players differs. Here, the abilities most frequently connected to competencies are muscle relaxation (3 occurrences), followed by joint flexibility and endurance in work (2), and movement speed and sustained effort capacity (1) are less dominant. This pattern can be explained by the sport-specific characteristics of handball. First, the game requires frequent explosive movements and sudden changes of direction, often accompanied by substantial body contact. Because the muscles are repeatedly subjected to high tension, the ability to relax them rapidly is crucial for both injury prevention and efficient recovery, which helps explain the strong associations observed with psychological competencies. Second, adequate joint flexibility facilitates swift directional changes and the absorption of impact during contact play, thereby establishing a functional relationship with the competencies necessary for high-level performance.

The analysis of correlation patterns reveals that the more frequent and numerous the connections between physical ability and competency, the more likely that physical factor plays a critical role in the development of competency. This relationship is not only statistically meaningful but functionally interpretable as well: physical abilities can be seen as significant “background factors” in the functioning of certain competencies. This supports the idea that competencies have a direct impact on sports performance. Various literatures also affirm that sports performance is not solely determined by physical attributes, but that mental and competency-based factors are key contributors to achieving excellence (32, 33) Recent empirical evidence also supports the role of psychological and competency-based factors in sports performance (34).

When the frequency of correlations is low or zero, physical abilities do not strongly support the functioning of competencies. Based on the current study, it can be concluded that in artistic gymnastics, extreme joint flexibility, while in handball. Single-effort capability, explosive power, dexterity, sustained speed are physical traits that do not significantly enhance competency function.

When the frequency of correlations is low or zero, physical abilities do not strongly support the functioning of competencies. Based on the current study, it can be concluded that in artistic gymnastics, extreme joint flexibility, while in handball, singular high-effort exertion are physical traits that do not significantly enhance competency function. This may also result from sport-specific characteristics, as excessive flexibility in gymnastics can compromise stability, while singular forceful exertions are less typical movement patterns in handball. In both sports, the technical level lies in the medium-frequency zone, indicating that while not a primary physical background factor, it still plays an incredibly significant role in the development of certain competencies.

Based on the above it can be stated that competencies, and consequently performance, are not solely influenced by physical factors, but also significantly by other variables. Physical ability “aspect” plays an important, albeit limited, role. Performance and overall effectiveness can be strongly affected by various psychological and mental processes. Some factors hinder, while others support final performance. For instance, motivation, beyond its energizing role, also serves a regulatory function by directing the athlete's behaviour, perseverance, and goal orientation. In contrast, anxiety, particularly when uncontrolled—may have a negative impact on performance. At the same time, self-confidence—as a positive psychological resource—can significantly enhance performance optimization (21, 35–38). The results confirm that different sports require distinct competent profiles (31). A more detailed examination of these factors is considered a key objective of our future research.

Although the pattern of significant correlations appears different between gymnastics and handball, these contrasts were not formally tested for statistical differences; therefore, they should be interpreted as descriptive and hypothesis-generating.

The small sample sizes (artistic gymnastics *N* = 14; handball *N* = 20) limit statistical power; therefore, these results should be interpreted cautiously as exploratory findings.

## 4 Discussion

The academic literature clearly highlights that outstanding athletic performance is complex construct, wherein psychological traits and competencies are closely interlinked with physical factors (22, 32, 33, 39). This exploratory pilot study examined the associations between twelve competencies and ten fundamental physical sub-skills in elite female artistic gymnasts and handball players.

Across the full sample, many competencies showed moderate-to-strong correlations with physical abilities (Table 2), supporting the idea that athletic competencies are at least partly grounded in physical capacities. These findings are consistent with multidimensional models of performance in which mental skills and physical preparation mutually reinforce one another (22, 33).

Among the aims of our current research was to explore the relationship between athletic competencies and their underlying physical sub-skills. Competencies area complex characteristic that plays a significant role in shaping performance. In our study, we examined the relationship between 12 competencies and 10 fundamental physical sub-skills. Our investigation focused on two key research questions: which physical abilities show an association with athletic competencies, and whether are differences in the competency patterns between two sports—artistic gymnastics and handball?

The method used in the research was a questionnaire-based data collection, whose results were evaluated by using statistical correlation analysis.

One of the most important findings of the analysis is that—particularly in the case of the artistic gymnasts—many competencies exhibit strong, interpretable, and multifaceted correlations with physical abilities, as evidenced by the correlation matrix (Table 2). More specifically, 4 out of the 12 competencies demonstrated a high frequency of correlations (in 5–6 instances), indicating significant relationships with physical attributes. Distinct sport-specific differences are readily apparent. These results partly support our first hypothesis (H1), which proposed that physical abilities are closely and consistently linked with athletic competencies. This outcome confirms that athletic performance is based on a multidimensional model in which both psychological and physical factors play fundamental roles (22, 33).

The comparison between the two sports also met expectations. The results confirmed our second hypothesis (H2), which suggested that the pattern of correlations between competencies and physical abilities would differ across sports (40). Although the pattern of correlations appeared different between gymnastics and handball, these contrasts were not formally tested for statistical differences; therefore, any sport-specific inferences remain descriptive and hypothesis-generating. Tentatively, gymnasts showed more frequent associations between endurance-type abilities and competencies such as perseverance, competition attitude, performance expectation, and satisfaction, whereas handball players tended to display links between speed-related abilities and competencies such as work ability and activity. These tendencies reflect the contrasting motor and cognitive demands of the two sports precision and sustained effort in gymnastics (41) vs. rapid decision-making and dynamic team interaction in handball (42).

The divergent correlations observed in the two samples clearly illustrate these differences. Among artistic gymnasts, the strongest correlations with physical abilities were found for the competencies of endurance, perseverance, activity, performance expectation, and satisfaction. These factors are crucial in artistic gymnastics, given the sport's emphasis on individual, static, and precisely executed movement sequences. During routines, continuous activity and physical presence are indispensable for successful performance (29, 41). Furthermore, for gymnasts, self-imposed performance expectations often serve as powerful internal motivation, ultimately reflected in a sense of satisfaction.

In contrast to the findings observed in gymnasts, the dominant competencies among handball players include work ability and activity. In team sports, most game situations require players to engage in continuous situational assessment, decision-making, and adaptation, which demand a high level of activity. For handball players, work ability is also essential for consistent and successful match performance, particularly under competitive conditions, and it plays a crucial role in ensuring long-term athletic development (24, 26).

The explanation for these differences can be traced back to the characteristic, functional, and structural features of the two sports. Artistic gymnastics is an individual sport that requires technically and physically complex movement sequences, in which endurance and perseverance play a fundamental role. The successful application of these two factors is not possible without sufficient physical engagement. In contrast, handball is a dynamic, interactive team sport. The ever-changing tasks and situations demand a high degree of focused attention. These findings collectively support the notion that the development of athletic competencies requires a sport-specific approach (26).

To enhance performance and effectiveness, it is recommended to focus on the development of competencies, especially those that, according to the results of this study, exhibit strong correlations with physical attributes. In competency development, both the continuous practice of specific skills and the enhancement of physical abilities appear to be effective tools.

## 5 Limitations

The small sample sizes (*N* = 14 and *N* = 20) limit the statistical power of our analyses, and the findings should therefore be regarded as exploratory. We did not implement multiple-comparison corrections for the 120 correlations because this investigation was designed as a pilot, hypothesis-generating study. Consequently, the reported relationships are preliminary and require confirmation in future research with larger samples.

Furthermore, although the pattern of significant correlations appears different between gymnastics and handball, these contrasts were not formally tested for statistical differences; thus, any sport-specific inferences remain descriptive and hypothesis-generating.

## 6 Conclusion

In conclusion, this exploratory study demonstrated that several athletic competencies exhibit sport-specific patterns of association with physical abilities among elite female artistic gymnasts and handball players. While endurance-related attributes were most strongly linked to competencies such as perseverance, performance expectations, and satisfaction in gymnastics, work ability and activity showed stronger connections to speed-related abilities in handball. These findings support the view that competencies are not generic constructs but are rooted in the motor and cognitive demands of each sport. Furthermore, the results underscore the importance of integrating both physical and psychological aspects in talent development and training programs. However, given the pilot nature and limited sample size, the correlations reported should be interpreted with caution. Future research with larger samples and comparative statistical models is needed to validate these sport-specific competency–ability profiles and inform evidence-based training interventions.

## Data Availability

The raw data supporting the conclusions of this article will be made available by the authors, without undue reservation.
